# Serum selenium concentration is associated with metabolic factors in the elderly: a cross-sectional study

**DOI:** 10.1186/1743-7075-7-38

**Published:** 2010-05-06

**Authors:** Kuen-Cheh Yang, Long-Teng Lee, Yow-Shan Lee, Hui-Ying Huang, Ching-Yu Chen, Kuo-Chin Huang

**Affiliations:** 1 Department of Family Medicine, National Taiwan University Hospital, 7 Chung-Shan South Road, Taipei, Taiwan; 2 Department of Family Medicine, Sijhih Cathay General Hospital, Taipei, Taiwan; 3 Department of Nutrition, College of Health Care, China Medical University, Taichung, Taiwan

## Abstract

**Background:**

Selenium is an essential micronutrient known for its antioxidant function. However, the association of serum selenium with lipid profiles and fasting glucose are inconsistent in populations with average intake of selenium. Furthermore, there were few studies conducted specifically for the elderly. This study examined the relationship of serum selenium concentration with serum lipids and fasting glucose in the Taiwanese elderly population.

**Methods:**

This was a cross-sectional study of 200 males and females aged 65-85 years (mean 71.5 ± 4.6 years) from Taipei, Taiwan. Serum selenium was measured by inductively coupled plasma-mass spectrometer. The association between serum selenium and metabolic factors was examined using a multivariate linear regression analysis after controlling several confounders.

**Results:**

The mean serum selenium concentration was 1.14 μmol/L, without significant difference between sexes. Total cholesterol, triglycerides, and LDL cholesterol increased significantly with serum selenium concentration (*P *< 0.001, *P *< 0.05 and *P *< 0.001, respectively) after adjusting for age, gender, anthropometric indices, lifestyle factors, and cardio-vascular risk factors in several linear regression models. Furthermore, there was a significantly positive association between serum selenium and serum fasting glucose concentrations (*P *< 0.05).

**Conclusions:**

Total cholesterol, triglycerides, and LDL cholesterol, and fasting serum glucose concentrations increased significantly with serum selenium concentration in the Taiwanese elderly. The underlying mechanism warrants further research.

## Background

Selenium, an essential trace element, is vital for human normal development, growth, male fertility, and thyroid hormone metabolism. Selenium deficiency is associated with Keshan disease, an endemic cardiomyopathy, and Kashin-Beck disease, a deforming arthritis. Less-overt deficiency may cause disease susceptibility and poor health maintenance [1]. Selenoproteins reportedly possess antioxidant properties and its deficiency plays an important role in the pathogenesis of cardiovascular disease (CVD). However, whether or not selenium concentration contributes to CVD remains uncertain. For example, epidemiologic studies show that low serum selenium is associated with increased cardiovascular mortality [2-4]. On the other hand, there is no significant correlation between selenium and risk of myocardial infarction [5] and peripheral artery disease [6] in selenium-replete population such as United States.

Some studies propose that selenium may exert its influence on CVD through its effect on known CVD risk factors, including dyslipidemia and hyperglycemia. Observational studies show positive associations of serum selenium with total cholesterol, low-density lipoprotein (LDL) cholesterol, and triglycerides concentrations in high selenium-replete populations [7-9]. However, in populations with low serum selenium concentrations, the associations are inconsistent [10-19]. Similarly inconsistent is the association between serum selenium and fasting glucose concentrations [7,20-22]. Potential adverse effects of high selenium exposure on diabetes risk have been noted [23-26]. Nonetheless, most studies involve relatively young populations. Few studies have focused on the elderly.

Since CVD is a leading cause of death in the elderly, the relationship between serum selenium concentrations and metabolic factors deserves further studies. This study aimed to evaluate the relationship between serum selenium and metabolic factors, including serum lipid and glucose concentrations, in a cohort of Taiwanese elderly.

## Methods

### Subjects

A total of two hundred ambulatory elderly volunteers living in the Taipei area were invited to our study in 2007. Information about age, sex, cigarette smoking, alcohol consumption, physical activity, CVD history, current use of cholesterol-lowering medications, current use of vitamin-mineral supplements, and current use of hormone replacement therapy were obtained by individual interview through questionnaires. Current smokers were defined as those with smoking recently for more than six months. Former smokers were defined as quitting smoking for at least one year. Former smokers and never smokers were grouped together as non-current smokers for further analysis. Current drinkers of alcohol were defined as those with drinking at least one ounce of alcohol per week for six months. Former drinkers were defined as quitting drinking for at least one year. Former drinkers and never drinker were grouped together as non-current drinker for further analysis. Current use of cholesterol-lowering medications was defined as participants using medication regularly. Current use of vitamin-mineral supplements was defined as participants taking supplements daily or at least weekly. Vitamin-mineral supplements included multivitamin, vitamin B complex, calcium supplement, glucosamine sulfate, collagen supplement and cod liver oil, etc. Current use of hormone replacement therapy was defined as taking hormone regularly. Anthropometric measurements, including height and weight, were performed using a standard stadiometer. Body mass index (BMI) was calculated as weight (kg) divided by height squared (m^2^). Diabetes mellitus (DM) and hypertension were defined based on self-reported history or current medication use for DM and hypertension.

### Blood analyses

Venous blood sample was taken after an eight-hour fasting at least. Serum glucose, total cholesterol, high-density lipoprotein (HDL) cholesterol, LDL cholesterol, and triglycerides were assessed by automatic spectrophotometric assay (HITACHI 7170, Japan). Fasting glucose was determined using glucose oxidase method. Using free glycerol banking method to determine serum triglycerides. HDL and LDL were determined by chemical modified enzyme and sodium N-(2-Hydroxy-3-sulfopropyl)-3.5-dimethoxyaniline (HSDA). Serum selenium was measured using inductively coupled plasma mass spectroscopy (ICP-MS). Serum samples were diluted 1:24 with diluents of 0.1% nitric acid and 0.1% Triton X-100. The calibration standards were prepared in a blank matrix and run using the standard addition calibration type. The serum samples were analyzed in the peak-jumping mode for ^82^Se, with the detection limit set at 0.01 μmol/L. Accuracy of the analysis was checked against Seronorm Trace Element Human Serum (batch 704121; Nycomed AS, Oslo, Norway) as reference material. The National Health Institute in Taiwan approved this study. All of the participants provided informed consents.

### Statistical analysis

Data were presented as means and standard deviations (mean ± SD) and percentages. Participants were divided into four groups according to their serum selenium concentrations. Anthropometric and biochemical variables were compared across the four quartiles. Tests for trend across serum selenium quartiles were calculated by entering the quartile as an ordinal number in a regression model. Using one-way analysis of variance, the study had a 83% power to detect the difference in means of total cholesterol levels with 50 subjects in each group and with an alpha of 0.05 characterized by a variance of 0.057, assuming that the common standard deviation is 0.970.

The relationship between serum selenium concentrations and lipids and fasting glucose were analyzed using several multivariate linear regression models with lipids and fasting glucose as dependent variables, and serum selenium as independent variable. Other possible confounders were adjusted for models 1~3 as independent variables. In model 1, age, sex and BMI were adjusted. In model 2, current smoking, current drinking, vegetarian diet, and physical activity were further adjusted. In model 3, hormone replacement therapy, cholesterol-lowering medication, vitamin supplement, diabetes mellitus, and hypertension were also further adjusted. For fasting glucose, a "modified" model 3 was applied after excluding diabetes mellitus from the independent variables in model 3. Log transformation of the variables was performed if they were not normally distributed as assessed by the Kolmogorov-Smirnov test. The least square (LS) means of lipids and glucose concentration were computed by general linear models adjusted for the independent variables among the four selenium quartile groups. Statistical analyses were performed using the SPSS statistical software (13^th ^version, SPSS Inc., Chicago, IL, USA).

## Results

The basic characteristics of the subjects were shown in Table 1. Their average age was 71.5 ± 4.6 years and mean serum selenium concentration was 1.14 ± 0.23 μmol/L. Serum total, LDL and HDL cholesterol were significantly higher in women (*P *< 0.001, *P *= 0.016 and *P *< 0.001, respectively) than in men but there was no significant difference in their selenium levels. However, fasting glucose was significantly higher in men than in women (*P *= 0.036). In Table 1, the means of total, LDL cholesterol, triglycerides, and fasting glucose concentrations were significantly different among the four selenium quartiles (*test for trend, P *= 0.0004, *P *= 0.02, *P *= 0.0008 and *P *= 0.04, respectively). In sex specific analyses, the means of triglycerides and fasting glucose concentrations increased significantly across the four selenium quartiles in men (*P *= 0.01 and *P *= 0.001, respectively) while the means of total, HDL and LDL cholesterol concentrations increased significantly across the four selenium quartiles (*P *< 0.0001, P = 0.028 and *P *< 0.0001, respectively) in women. The characteristics among the four selenium quartiles separately for men and women were shown in additional file 1, Table S1 and Table S2, respectively.

**Table 1:** General characteristics among quartiles of serum selenium concentrations

	Q1 (n = 50)	Q2 (n = 51)	Q3 (n = 49)	Q4 (n = 50)	
	<0.98 μmol/L	0.98-1.136 μmol/L	1.14-1.30 μmol/L	>1.30 μmol/L	*P for trend*^a^
Age (y)	71.8 ± 5.2	70.1 ± 3.9	71.6 ± 4.8	72.5 ± 4.4	0.235
Sex, male (%)	26	27.5	34.7	38	0.141
Body weight (kg)	57.7 ± 10.7	58.3 ± 9.8	57.7 ± 8.6	56.2 ± 10.1	0.521
WC (cm)	82.4 ± 10.6	82.3 ± 8.9	82.2 ± 8.3	80.1 ± 10.3	0.253
BMI (kg/m^2^)	23.9 ± 3.7	23.7 ± 3.1	23.5 ± 2.6	22.6 ± 3.2	0.049
TCHO (mmol/L)	5.06 ± 0.83	5.27 ± 0.88	5.38 ± 1.03	5.72 ± 1.01	0.0004
LnTG (mmol/L)	0.1 ± 0.44	0.2 ± 0.49	0.29 ± 0.46	0.31 ± 0.51	0.002
HDL-C (mmol/L)	1.44 ± 0.27	1.49 ± 0.37	1.5 ± 0.46	1.52 ± 0.35	0.271
LDL-C (mmol/L)	3.01 ± 0.69	3.1 ± 0.64	3.2 ± 0.7	3.47 ± 0.71	0.0008
Sugar-AC (mmol/L)	5.34 ± 0.73	5.68 ± 1.28	5.63 ± 0.76	5.84 ± 1.45	0.04
Se (μmol/L)	0.85 ± 0.09	1.07 ± 0.04	1.22 ± 0.05	1.44 ± 0.1	<0.0001
Current smoking (%)	4	9.8	6.1	6.1	0.9
Current drinking (%)	14	17.6	16.3	10.0	0.547
HTN (%)	48	45.1	49	44	0.796
DM (%)	8	11.8	20.4	8	0.7
Lipid Tx (%)	22	17.6	20.4	24	0.732
Vegetarian (%)	4	2	0	6	0.703
Exercise (min/week)	340.6 ± 199.3	334.8 ± 224.5	322.8 ± 240.7	333.0 ± 239.2	0.808
					
Vitamin-mineral supplement users (%)	78	84.3	85.7	90	0.102

Abbreviations: WC, waist circumference; TCHO, total cholesterol; LnTG, log transformation of triglycerides; HDL-C, high-density lipoprotein cholesterol; LDL-C, low-density lipoprotein cholesterol; Se, serum selenium; Sugar-AC, fasting serum glucose; HTN, hypertension; DM, diabetes mellitus; Lipid Tx, hyperlipidemia treatment; HRT, hormone replacement treatment.

^a^*P value for trend *in percentages or means across quartiles of serum selenium

Serum selenium concentration was positively associated with serum total, LDL cholesterol, and triglycerides using multivariate linear regression analyses adjusting for various confounders in Models 1-3 in Table 2. Serum fasting glucose was also positively associated with serum selenium in Model 1, Model 2 and "modified" Model 3 (*P *= 0.026, *P *= 0.034 and *P *= 0.03, respectively). The result of sex-specific analyses was provided in additional file 1, Table S3 and Table S4.

**Table 2:** Linear regression models showing standardized betas with serum selenium concentrations as independent variable

	TCHO		LnTG		HDL-C		LDL-C		Sugar-AC	
Model	Beta	P-value	Beta	P-value	Beta	P-value	Beta	P-value	Beta	P-value
Model 1	0.276	<0.001	0.194	0.006	0.086	0.204	0.259	<0.001	0.157	0.026
Model 2	0.275	<0.001	0.189	0.007	0.086	0.185	0.257	<0.001	0.15	0.034
Model 3	0.294	<0.001	0.183	0.012	0.093	0.173	0.279	<0.001	0.156^a^	0.03^a^

Abbreviations: TCHO, total cholesterol; LnTG, log transformation of triglycerides; HDL-C, high-density lipoprotein cholesterol; LDL-C, low-density lipoprotein cholesterol; Se, serum selenium; Sugar-AC, fasting serum glucose

Model 1: adjusted for age, sex, and BMI

Model 2: adjusted for age, sex, BMI, current smoking, current drinking and vegetarianx diet, and physical activity

Model 3: adjusted for age, sex, BMI, current smoking, current drinking and vegetarian diet, and physical activity, hormone replacement therapy, cholesterol-lowering medication, Vitamin supplement, diabetes mellitus, hypertension

^a ^"Modified" Model 3: Diabetes mellitus was excluded in Model 3

**Figure 1:**
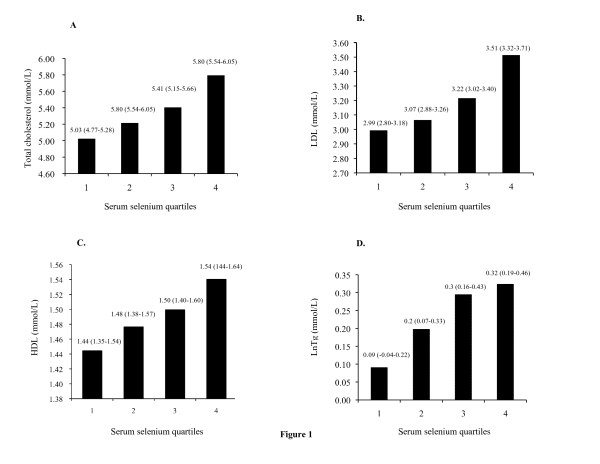
**Relationship between serum selenium and total (A), LDL (B), HDL cholesterol (C), and triglycerides (D) levels**. The least square (LS) means of serum lipid in the quartiles of serum selenium (<0.98 μmol/L, 0.98-1.136 μmol/L, 1.14-1.3 μmol/L, >1.3 μmol/L in serum selenium) using general linear model after adjustment for confounders in model 3. Covariates appearing in the models were estimated with the following values: age = 71.4968, sex = 0.69, BMI = 23.423, current smoking = 0.065, current drinking = 0.145, vegetarian diet = 0.03, physical activity = 332.85, hormone replacement therapy = 0.18, cholesterol-lowering medication = 0.21, vitamin supplement = 0.77, diabetes mellitus = 0.12, hypertension = 0.465. The LS means of total cholesterol increased with the increments of serum selenium (*P for trend *< 0.001); the LS means of log transformation of triglycerides increased with increments of serum selenium (*P for trend *< 0.01); the LS means of LDL cholesterol increased with increments of serum selenium (*P for trend *< 0.001), and the LS means of HDL cholesterol increased with increments of serum selenium (*P for trend *= 0.152).

The LS means of total, LDL, HDL cholesterol, and triglycerides in the four selenium quartiles were shown in Figure 1. The LS means of total (Figure 1A), LDL cholesterol (Figure 1B), and triglycerides (Figure 1D) increased significantly with increments of serum selenium quartiles. However, no significant difference was found in the LS means of HDL cholesterol (Figure 1C) with increments of serum selenium.

## Discussion

In this study, serum selenium concentrations were positively associated with serum concentrations of total cholesterol, LDL cholesterol, triglycerides, and glucose in the elderly, after adjustment for age, gender, anthropometric indices, lifestyles, and traditional CVD risk factors. This suggests that high serum selenium concentrations may be associated with risk factors of CVD in the elderly.

Positive relationships between serum selenium and total cholesterol concentrations are discussed in several studies of various serum selenium concentrations [2,4,7,10-15,19,27]. However, most participants have been young and middle-aged adults. Similar to two studies involving the elderly [4,14,15], this study showed that serum total cholesterol concentration was positively associated with serum selenium concentration (Table 2; Fig. 1A).

Many studies failed to show a significant association between serum selenium and triglycerides concentrations [10,12,27] and only two have shown a positive association [7,8]. Our study demonstrated that serum selenium concentrations were positively associated with serum triglycerides (Table 2; Fig. 1D).

The association between serum selenium and LDL cholesterol levels was not consistent in previous studies. Five studies showed a positive association [7-9,13,14] but others revealed no significant relationship [11,16-18]. The variable sample sizes, age groups, and lack of adjustments for possible confounders may account for the inconsistencies in those studies. Our present study supported that serum LDL cholesterol concentrations were positively associated with serum selenium levels (Table 2; Fig. 1B).

The association of serum selenium with HDL cholesterol is not consistent in previous studies. Five studies report a positive association [8,9,11,15,16], one has a negative association [7], and three do not show any significant association [10,12,19]. In our present study, the HDL cholesterol concentrations did not increase significantly with increments of serum selenium (Table 2; Fig. 1C). In addition, the mean HDL cholesterol concentration was higher in our population as compared to those in previous studies.

Interestingly, the effects of selenium supplementation on blood lipids are contradictory in animal and human studies. In rats, selenium supplementation increases LDL receptor activity [28,29] but decreases 3-hydroxy 3-methylglutaryl co-enzyme A (HMG-CoA) reductase expression [30], leading to decreased plasma LDL cholesterol and total cholesterol levels. However, one animal study in mice showed a significant increase in plasma cholesterol with the loss of housekeeping selenoprotein expression [31]. In human, selenium supplementation was found to increase total cholesterol and triglyceride levels in French adults [27]. Total cholesterol and LDL cholesterol levels also increased after selenium supplementation in the Chinese population [32]. Another study showed no further decrease in triglyceride or LDL cholesterol concentration but a blunted increment of HDL with selenium supplementation in participants with coronary heart disease receiving simvastatin-niacin treatment [33]. Therefore, the role of selenium supplementation on lipid metabolism in humans deserves further research. Recently, the apoE δ4 gene was found to play a central role between selenium levels and lipid metabolism in rural elderly Chinese [34]. The underlying interactive mechanism between susceptible gene, selenium, and lipids needs further investigation.

High serum selenium concentrations also correlate with high fasting glucose concentrations [7,20]. The prevalence of type 2 diabetes increases with increment of serum selenium in American adults [23]. Further analysis of the study shows that glycosylated hemoglobin levels increase with increasing selenium concentrations [26]. Similarly, serum selenium level was positively associated with glucose level in Table 2. Stranges *et al *note increased risk of diabetes after receiving selenium supplements for 7.7 years [24]. Lipman *et al *also found a statistically non-significant increase in diabetes after selenium supplementation for 5.46 years [25]. Interestingly, over-production of selenium-dependent glutathione peroxidase-1 can induce hyperinsulinemia in mice [35]. In contrast, Hughes *et al *showed no significant difference in serum selenium levels between diabetic and non-diabetic Singaporeans [36]. Recently, Akbaraly *et al *found that higher serum selenium status at baseline had protective effect on later occurrence of dysglycemia in a 9-year longitudinal study [22]. Besides, supplementation of selenium can lead to increased insulin sensitivity in rats [37]. Therefore, the inconsistent results of various studies imply that further research is warranted.

Although this study shows that elevated serum selenium concentrations are associated with metabolic factors in an elderly population, it has some limitations. First, the study has a cross-sectional design and causal inference cannot be established. Second, the participants are volunteers rather than randomly selected. External validation is necessary in future studies. In addition, there is no human selenium survey in Taiwan. Thus, a comparison of serum selenium concentrations between the data here and the Taiwanese data is not possible. Thirdly, there is no available data on food intake and selenium supplementation, which may indicate a possible residual confounding effect. Fourth, multivariate analyses adjusted for lipid-lowering medications might bias the results [38]. Finally, only the serum fasting glucose without insulin levels was available in the study. Therefore, the relationship between selenium with insulin resistance could not be explored.

## Conclusions

In conclusion, serum selenium concentration was associated with serum concentrations of total, LDL cholesterol, triglycerides, and glucose in the elderly after adjustment for potential confounders. The role of selenium on lipid and glucose metabolism in humans deserves further research in the future. Thus, recommendations on selenium supplementation should be carefully evaluated.

## Competing interests

The authors declare that they have no competing interests.

## Authors' contributions

KCY was in charge of data analysis and writing of the draft. LTL, CYC participated in the data collection and study design. HYH was responsible for serum selenium measurement. YSL helped with the data management and interpretation of results. KCH synthesized the analyses and headed the writing of the manuscript. All of the authors read and approved the final manuscript.

## Supplementary Material

Additional file 1 **General characteristics by quartiles of serum selenium in men and women**. The characteristics among the four selenium quartiles separately for men and women were shown in Table S1 and Table S2, respectively. **Linear regression models showing standardized coefficients with serum selenium concentrations as independent variable in men and women**. The analyses of multivariate linear regression models for men and women were shown in Table S3 and Table S4, respectively. Click here for file
