# Supplementary material for: Serum selenium concentration is associated with metabolic factors in the elderly: a cross-sectional study
Source: Nutr Metab (Lond). 2010 May 6;7:38. doi: 10.1186/1743-7075-7-38 (PMC2873298; doi:10.1186/1743-7075-7-38)
Supplement: Additional file 1 — General characteristics by quartiles of serum selenium in men and women. The characteristics among the four selenium quartiles separately for men and women were shown in Table S1 and Table S2, respectively. Linear regression models showing standardized coefficients with serum selenium concentrations as independent variable in men and women. The analyses of multivariate linear regression models for men and women were shown in Table S3 and Table S4, respectively. [file 1743-7075-7-38-S1.DOC]

|  | Q1 (n=13) | Q2 (n=14) | Q3 (n=17) | Q4 (n=19) | *p for trend*^a^ |
| --- | --- | --- | --- | --- | --- |
|  | <0.98 µmol/L | 0.98-1.136 µmol/L | 1.14-1.30 µmol/L | >1.30 µmol/L |  |
| Age (y) | 74.3±4.8 | 73.2±4.4 | 75.2±5.0 | 74.0±3.4 | 0.868 |
| Body height (cm) | 162.2±5.1 | 164.3±5.5 | 165.0±5.2 | 164.4±6.3 | 0.303 |
| Body weight (kg) | 63.1±13.6 | 66.7±8.2 | 63.2±8.4 | 64.5±9.7 | 0.98 |
| WC (cm) | 86.6±13.7 | 89.6±7.6 | 85.5±8.6 | 86.9±10.0 | 0.748 |
| BMI (kg/m^2^) | 23.9±4.2 | 24.7±3.3 | 23.3±2.7 | 23.9±3.5 | 0.704 |
| TCHO (mmol/L) | 5.0±0.7 | 5.0±0.74 | 4.66±0.83 | 5.35±0.98 | 0.274 |
| LnTG (mmol/L) | -0.19±0.36 | 0.33±0.50 | 0.37±0.41 | 0.33±0.59 | 0.01 |
| HDL-C (mmol/L) | 1.45±0.21 | 1.32±0.35 | 1.22±0.25 | 1.38±0.26 | 0.42 |
| LDL-C (mmol/L) | 3.03±0.58 | 2.94±0.52 | 2.80±0.70 | 3.27±0.78 | 0.342 |
| Sugar-AC (mmol/L) | 5.24±0.43 | 5.44±0.62 | 5.79±0.84 | 6.67±2.02 | 0.001 |
| Se (µmol/L) | 0.84±1.0 | 1.07±0.04 | 1.22±0.05 | 1.43±0.08 | < 0.0001 |
| Current smoking (%) | 15.4 | 35.7 | 11.8 | 15.8 | 0.565 |
| Current drinking (%) | 30.8 | 35.7 | 35.3 | 21.1 | 0.512 |
| HTN (%) | 61.5 | 42.9 | 52,9 | 47.4 | 0.598 |
| DM (%) | 0 | 14.3 | 41.2 | 15.8 | 0.158 |
| Lipid Tx (%) | 15.4 | 21.4 | 5.9 | 26.3 | 0.62 |
| Vegetarian (%) | 0 | 0 | 0 | 0 | - |
| Exercise (min/week) | 358.5±198.1 | 386.4±140.2 | 330.0±247.6 | 317.9±243.2 | 0.786 |
| Vitamin-mineral supplement users (%) | 76.9 | 78.6 | 70.6 | 68.4 | 0.498 |

**Table S1.** General characteristics by quartiles of serum selenium in men

**Table S2. General characteristics by quartiles of serum selenium in women**

|  | Q1 (n=37) | Q2 (n=37) | Q3 (n=32) | Q4 (n=31) | *p for trend*^a^ |
| --- | --- | --- | --- | --- | --- |
|  | <0.98 µmol/L | 0.98-1.136 µmol/L | 1.14-1.30 µmol/L | >1.30 µmol/L |  |
| Age (y) | 71.0±5.1 | 68.9±3.0 | 69.7±3.4 | 71.6±4.7 | 0.495 |
| Body height (cm) | 152.5±6.0 | 153.2±6.3 | 152.0±4.6 | 153.2±5.6 | 0.853 |
| Body weight (kg) | 55.4±8.8 | 55.1±8.4 | 54.7±7.2 | 51.2±6.3 | 0.039 |
| WC (cm) | 80.92±9.0 | 79.6±7.8 | 80.5±7.7 | 76.0±8.3 | 0.035 |
| BMI (kg/m^2^) | 23.8±3.5 | 23.4±2.9 | 23.7±2.5 | 21.8±2.6 | 0.016 |
| TCHO (mmol/L) | 5.09±0.88 | 5.39±0.91 | 5.78±0.91 | 5.96±0.97 | < 0.0001 |
| LnTG (mmol/L) | 0.21±0.41 | 0.15±0.49 | 0.25±0.48 | 0.30±0.46 | 0.325 |
| HDL-C (mmol/L) | 1.44±0.29 | 1.55±0.37 | 1.65±0.48 | 1.62±0.36 | 0.028 |
| LDL-C (mmol/L) | 3.0±0.74 | 3.16±0.67 | 3.41±0.61 | 3.59±0.65 | < 0.0001 |
| Sugar-AC (mmol/L) | 5.38±0.82 | 5.76±1.46 | 5.55±0.71 | 5.33±0.52 | 0.684 |
| Se (µmol/L) | 0.85±0.09 | 1.07±0.04 | 1.22±0.05 | 1.44±0.1 | < 0.0001 |
| Current smoking (%) | 0 | 0 | 1 | 0 | 0.61 |
| Current drinking (%) | 3 | 4 | 2 | 1 | 0.355 |
| HTN (%) | 43.2 | 45.9 | 46.9 | 41.9 | 0.953 |
| DM (%) | 10.8 | 10.8 | 9.4 | 3.2 | 0.283 |
| Lipid Tx (%) | 24.3 | 16.2 | 28.1 | 22.6 | 0.84 |
| Vegetarian (%) | 5.4 | 2.7 | 0 | 9.7 | 0.574 |
| HRT | 27 | 29.7 | 21.9 | 25.8 | 0.73 |
| Exercise (min/week) | 334.3±202 | 353.1±248.2 | 318.9±240.8 | 342.3±240.3 | 0.95 |
| Vitamin-mineral supplement users (%) | 70.3 | 73 | 81.3 | 93.5 | 0.016 |

Abbreviations: WC, waist circumference; TCHO, total cholesterol; LnTG, log transformation of triglycerides; HDL-C, high-density lipoprotein cholesterol; LDL-C, low-density lipoprotein cholesterol; Se, serum selenium; Sugar-AC, fasting serum glucose; HTN, hypertension; DM, diabetes mellitus; Lipid Tx, hyperlipidemia treatment; HRT, hormone replacement treatment.

^a^*P* value for trend in percentages or means across quartiles of serum selenium

**Table S3.** Linear regression models showing standardized coefficients with serum selenium concentrations as independent variable in men

|  | TCHO | | LnTG | | HDL-C | | LDL-C | | Sugar-AC | |
| --- | --- | --- | --- | --- | --- | --- | --- | --- | --- | --- |
| Model | Beta | P-value | Beta | P-value | Beta | P-value | Beta | P-value | Beta | P-value |
| Model 1 | 0.171 | 0.176 | 0.325 | 0.006 | -0.082 | 0.512 | 0.150 | 0.236 | 0.428 | 0.001 |
| Model 2 | 0.157 | 0.218 | 0.320 | 0.008 | -0.090 | 0.470 | 0.136 | 0.288 | 0.413 | 0.001 |
| Model 3 | 0.171 | 0.173 | 0.289 | 0.021 | -0.097 | 0.464 | 0.156 | 0.213 | 0.377^a^ | 0.002^a^ |

Abbreviations: TCHO, total cholesterol; LnTG, log transformation of triglycerides; HDL-C, high-density lipoprotein cholesterol; LDL-C, low-density lipoprotein cholesterol; Se, serum selenium; Sugar-AC, fasting serum glucose

Model 1: adjusted for age, sex, and BMI

Model 2: adjusted for age, sex, BMI, current smoking, current drinking and vegetarianx diet, and physical activity

Model 3: adjusted for age, sex, BMI, current smoking, current drinking and vegetarian diet, and physical activity, hormone replacement therapy, cholesterol-lowering medication, Vitamin supplement, diabetes mellitus, hypertension

^a^ “Modified” Model 3: Diabetes mellitus was excluded in Model 3

**Table S4. Linear regression models showing standardized coefficients with serum selenium concentrations as independent variable in women**

|  | TCHO | | LnTG | | HDL-C | | LDL-C | | Sugar-AC | |
| --- | --- | --- | --- | --- | --- | --- | --- | --- | --- | --- |
| Model | Beta | P-value | Beta | P-value | Beta | P-value | Beta | P-value | Beta | P-value |
| Model 1 | 0.313 | <0.001 | 0.096 | 0.278 | 0.150 | 0.084 | 0.292 | 0.001 | 0.01 | 0.905 |
| Model 2 | 0.310 | <0.001 | 0.091 | 0.298 | 0.156 | 0.07 | 0.287 | 0.001 | 0.003 | 0.973 |
| Model 3 | 0.299 | <0.001 | 0.063 | 0.498 | 0.183 | 0.046 | 0.269 | 0.004 | -0.085^a^ | 0.932^a^ |

Abbreviations: TCHO, total cholesterol; LnTG, log transformation of triglycerides; HDL-C, high-density lipoprotein cholesterol; LDL-C, low-density lipoprotein cholesterol; Se, serum selenium; Sugar-AC, fasting serum glucose

Model 1: adjusted for age, sex, and BMI

Model 2: adjusted for age, sex, BMI, current smoking, current drinking and vegetarianx diet, and physical activity

Model 3: adjusted for age, sex, BMI, current smoking, current drinking and vegetarian diet, and physical activity, hormone replacement therapy, cholesterol-lowering medication, Vitamin supplement, diabetes mellitus, hypertension

^a^ “Modified” Model 3: Diabetes mellitus was excluded in Model 3
